# 

**Published:** 1912-09-14

**Authors:** 


### Obituary.

Dr. John Edward Meredith, of Maidstone, has died after a long illness at St. Leonards. He was a very familiar figure in the Kentish county town, being public vaccinator and district medical officer. Dr. Meredith served for many years as honorary physician to the West Kent General Hospital, Maidstone, and at the time of his death he was consulting physician to this institution. He received his medical education at Trinity College, Dublin, where he qualified in 1872, later taking the degrees of M.D. and M.Ch.

Dr. Harold Charles Lingham has died at Herne Hill, S.E. He studied at St. Bartholomew's Hospital, and graduated in medicine, surgery, and science at the University of Edinburgh. His father was a medical practitioner at Birmingham.

Dr. Andrew Wilson, of lay newspaper fame, lecturer on physiology and health to the George Combe Trust and Gilchrist Trust lecturer, has died suddenly at North Berwick. Born in Edinburgh in September 1852, he was educated at Edinburgh University. In 1876 he was appointed lecturer at the Edinburgh Medical School on zoology and comparative anatomy, and on these and kindred subjects he wrote a number of books and magazine articles.

Dr. Charles Edward Hutt has died at Tottenham at the age of forty-seven. Dr. Hutt was very well known and one of the hardest workers in the British Medical Association. He was chairman of the North Middlesex Division in 1905, and to his efforts is mainly due the present standing of this division. Dr. Hutt qualified from St. Bartholomew's Hospital in 1888 at the Conjoint Board. He was at one time resident medical officer at the Hertford Infirmary. Since 1896 he had been in practice in Tottenham, where he was universally held in the highest esteem.

Sir William J. Sinclair, Professor of Obstetrics and Gynæcology at Victoria University, died last week at the age of sixty-six. Sir William was educated at Aberdeen University, where he qualified in 1873. Thence he went to be resident medical officer at St. Mary's Hospital, Manchester, to which he afterwards became physician. In the meanwhile he studied obstetrics in Vienna, after which he returned to Manchester. The list of his appointments and contributions to medical literature is a lengthy one; perhaps the most notable of his literary activities were his editorship of the *Medical Chronicle* and the establishment ten years ago of the *Journal of Obstetrics and Gynæcology of the British Empire.* Sir William Sinclair was knighted in 1904.

Surgeon-General Duncan Alexander Campbell Fraser has died at Cheltenham at the age of eighty. Afer taking his medical degree at Edinburgh in 1853 he served for nearly forty years in the Army Medical Service, his Staff appointments including the medical commands at Netley and Malta. He held the Star of Roumania for services as a Commissioner of the Red Cross Society in the Russo-Turkish war.

### BUREAU OF INFORMATION.

### Rules for Correspondents.

1. Every letter, on whatever subject, **must be accompanied by the coupon** to be cut from the back cover (inside page) of The Hospital, current issue, and must contain the name and address of the correspondent with pseudonym for publication if desired.

2. Replies by post cannot be given, save under exceptional circumstances at the Editor's discretion.

3. Letters from Approved Homes in reply to special needs published in the Bureau should state terms and full particulars, and be sent prepaid under cover to the Editor of the Bureau with coupon and name enclosed for identification.

4. Proprietors of Homes which have not yet been entered on the List of Approved Homes, but have spare accommodation likely to suit special needs, are invited to write for an application form for registration. The fee for registration, which includes two announcements of the Home in the Bureau, is 5s.

5. Special needs of every description relating to those who are sick or in difficulties are made known in these columns **free of charge.**

6. All communications to be addressed to The Editor of The Hospital, 28 Southampton Street, Strand, London, W.C., and marked "Bureau of Information."

### Training and Employment.

**Nursing Institutes and Percentages.**—The best nursing co-operations charge $7\frac{1}{2}$ per cent. on the nurses' earnings, and do very well with this on account of the numbers and the fairly constant nature of the work. Smaller institutes no doubt find it hard to pay their way under a 10 per cent. charge, which amounts to 2s. $1\frac{1}{4}$d. on each guinea. The expenses include the maintenance of a night and day service, with two secretaries, rooms in good situation, telephone, etc. If you think it out, you will see that this mounts up to at least £250 a year, however economically managed. Towards this you contribute, if you are engaged for forty weeks in the year at £2 2s. a week, the sum of £8 8s., and, unless there are at least thirty of you similarly employed, the institute would be working at a loss. Few private nurses realise the expenses inevitable to a supply of nurses.—L. M.

**Bureau of Employment.**—We are very glad to make it known that you are willing to receive applications for employment from Nurse W. and others, on the sole condition that they are members of the Royal National Pension Fund for Nurses. We note that "the object of the Bureau is to bring into touch the employers seeking reliable, capable nurses for permanent or chronic cases and those nurses desiring such cases, without any fee on either side." It is highly satisfactory that ninety-five engagements were entered into last year.—Miss Rosa Smith, Secretary, 15 Buckingham Street, Strand, W.C.

**Trained Children's Nurses.**—Both the Royal Devon and Exeter Hospital and also the South Devon Hospital at Plymouth could send you a trained children's nurse, if required. If you want, however, a "nursery nurse," apply to the Norland Institute, 10 Pembridge Square, London, W.—Mrs. F. W. (Winkfield).

**Massage Tuition.**—See The Hospital Bureau of July 20. Hospitals giving a course of massage to outside students are the West End Hospital, 73 Welbeck Street, London; the National Hospital, Queen's Square, Bloomsbury; and the Hospital for Epilepsy and Paralysis, Maida Vale, W. We do not know of any such facilities in Yorkshire Hospitals, and shall be much obliged if one of our correspondents will give us information on the subject.—E. H. (Sheffield).

**Sanatorium Posts.**—As you are already working in a sanatorium you are in a good position for procuring employment in the same line. We can only recommend you to wait a while, as districts are not yet mapped out. The principal medical papers are the ones to study.—Mr. C. O. N.

**School for Domestics.**—We recommend you to apply on behalf of your daughter to the Matron, Guy's Hospital, S.E. The school at Guy's is admirably managed, and affords a good career to any who enter it, as the girls are encouraged to attend classes and are prepared for taking good posts after the conclusion of their term of service.—T. C.

**Matron's Post Wanted.**—You are certainly not too old at forty-six to take a matronship at a convalescent home, school sanatorium, etc., and your previous experience in housekeeping and superintendence will stand you in good stead. Have your testimonials typed, and answer every advertisement you see which is likely to suit. It is entirely a question of perseverance. It is useless to aim at the big institutions under the circumstances, as competition is too severe.—E. A. C.

**Post as Nurse Companion.**—See reply to Sister Rosa Smith. She might be able to help you, but we regret we are unable to do so.—X. Y.

### Approved Homes.

Note.—*No Home is added to the List without direct medical and other testimony to its good management.*

**High-Class Nursing Home in London.**—23 Westbourne Gardens, Bayswater, London, W. Proprietors: Miss Watson and Miss Mudd. Excellent accommodation for medical, surgical, maternity, rest cure, and chronic patients. Personal supervision. Special attention to good dieting. Quiet locality.

### Needs and Helpers.

**After-Care for Phthisical Patient.**—We are pleased to make it known that you are seeking for a home for a lad, formerly clerk in an office, who is recovering from phthisis after some months' treatment. As payment of from 10s. to 12s. can be made for him, it ought not to be impossible to find him a home where he could have companionship and some work to do towards his maintenance. We note he is a Roman Catholic. Prepaid letters forwarded to M. W. (121z).

### Miscellaneous.

**The Guild of St. Barnabas.**—Write for full particulars to the Secretary-General, Church House, Westminster. All trained nurses and midwives who are members of the English Church are eligible for membership. There are branches in many different localities in England, and also in India and the Colonies. The members of the Guild meet periodically for devotional purposes, and a marked feature of the organisation is the spirit of good-fellowship one to another which it develops among those who belong to it. Members are entitled to wear the badge. Can one of our readers give us information about the "Guild of St. John of Jerusalem?"—Nurse G.

### Letter-Box.

D. B.—We are sorry we cannot undertake to forward the desired address or to recommend this particular form of treatment, but we thank you for suggestion.

Miss E. R. B.—We are very pleased to know that the announcement of your home in these columns brought you an immediate application from a patient. We are making inquiries into the agency you name, and will let you know the result. The preliminary fee is exorbitant.

E. H. B. (118a).—Very glad to know that one of our Approved Homes has been able to take your aunt.

### Gifts and Bequests.

Mr. Tregonwell Monro, of Cranborne, Dorset, has left £200 each to the Royal Victoria Hospital, Bournemouth, and the Salisbury Hospital, and £100 to King Edward's Hospital Fund for London.

Mrs. Drew, of Ardencaple House, Row, Dumbartonshire, has left the following public bequests, free of legacy duty and bearing interest from six months after death:—£2,000 to Helensburgh Infirmary, to endow a bed to be called the Rosina Douglas bed; and £1,000 each to Glasgow Royal Infirmary, Glasgow Western Infirmary, and Glasgow Victoria Infirmary.

Mr. Edward O'Connor Terry, of Priory Lodge, Barnes, S.W., the actor, who died on April 2, aged sixty-seven, leaving £44,056, has bequeathed £100 each to the Foundling Hospital Benevolent Fund, the Charing Cross Hospital, and the League of Mercy. He directed that the legacies for charitable purposes are only to be paid in the event of his estate being more than sufficient to pay the other legacies.

Mr. David Quixano Henriques, of Sussex Square, W., whose estate has been sworn for probate at £126,480, has bequeathed £500 to the Jews' Hospital and Orphan Asylum at West Norwood, to form the nucleus of a fund for the benefit of former beneficiaries of the institution; £200 to King Edward's Hospital Fund; £100 to St. Mary's Hospital, Paddington. He declared that the charitable legacies were few and of small amount, because he had throughout his life been a contributor to charity.

Mr. Herbert James Garrod, of Cheveley, near Newmarket, has left £200 to Addenbrooke's Hospital, Cambridge.

Miss Fanny Back, of St. Leonards-on-Sea, has bequeathed £400 to the Hospital for Consumption and Diseases of the Chest, London.

Mr. Henry Schlesinger, of Eaton Place, S.W., who left estate of the gross value of £141,083, has bequeathed £100 to St. Mary's Hospital, Paddington.

A welcome gift of luxuries for the benefit of patients in the Royal Free Hospital has been received from Queen Amelia of Portugal as a souvenir of her recent visit.

Mr. Henry Wells Smart, of Highgate, N., late manager of the London City and Midland Bank (Limited), has left £150 to the Hospital for Sick Children, Great Ormond Street.

Mr. Peter MacKellar, of Greenock, feuar, has bequeathed £3,000 each to the Greenock Hospital and Infirmary, to endow three beds, and £2,000 for the Manchuria medical work and mission.

Mr. William Peart, a former Master of the Leathersellers' Company, has bequeathed £300 to the endowment fund of the Tottenham Hospital and £100 to the Tottenham and Edmonton General Dispensary.

Dr. Theodore Leighton Pennell, M.D., F.R.C.S., of Bannu, on the North-West Frontier of India, medical missionary under the Church Missionary Society, who left estate valued at £25,202 gross, has left £1,000 to the Salvation Army Mission Hospital at Anand, Gujerat.

Mr. Rowland Swann, of Buxton, farmer, after making a number of bequests, has left all other his property to his wife, Mrs. Henrietta Swann, for life, and on her decease £1,000 each to the Devonshire Hospital and Bath Charity, Buxton, and the Buxton and District Cottage Hospital at Sherbrook.

Mr. Thomas Henry Keesing, of Regency Square, Brighton, whose instructions to his executors enjoined elaborate safeguards against being buried alive, has bequeathed £1,000 each to the Jewish Hospital and Orphan Asylum, Norwood, and the Jewish Home for Incurables, South Tottenham, for the naming of four beds; £500 to Lady Judith Montefiore Home for Convalescent Jews at St. Patrick's Road, Hove; and £100 to the Sussex County Hospital.

### Appointments.

Dr. Donaldson Robert, M.A., M.B., Ch.B.Edin., F.R.C.S.Edin., D.T.M., D.P.H., has been appointed pathologist and bacteriologist to the Royal Berkshire Hospital.

As was anticipated last week, the Brighton Town Town Council has appointed Dr. Duncan Forbes, medical officer of health, chief tuberculosis officer for the borough for the purposes of the Insurance Act. Four consumptive patients in the borough sanatorium are receiving treatment under the Act.

The King has been pleased, on the recommendation of the Secretary for Scotland, to approve the appointment of Robert Gordon McKerron, M.A., M.D., to be Professor of Midwifery in the University of Aberdeen in the place of Professor William Stephenson, who has resigned. Dr. McKerron, who is a graduate of Aberdeen, was formerly senior physician at the Aberdeen Children's Hospital and physician to the Maternity Hospital of the city. His publications include "Pregnancy, Labour, and Childbed with Ovarian Tumour."

